# Challenging the extended phenotype: HRD-negative salivary gland carcinoma in a *BRCA1* founder-variant carrier, case report and literature review

**DOI:** 10.3389/fonc.2025.1692001

**Published:** 2026-01-21

**Authors:** William Torres, Elizabeth Vargas, Diego-Felipe Ballen, Sandra M Tapiero-Rodriguez, Enrique Cadena, Rafael Parra-Medina, Julian C Riaño-Moreno

**Affiliations:** 1 Department of Pathology and Molecular Oncology, Instituto Nacional de Cancerología, Bogotá, D.C, Colombia; 2 Career Researcher, Hospital Universitario Mayor-Méderi, Universidad del Rosario, Bogotá, D.C, Colombia; 3 Clinical Oncology Unit, Instituto Nacional de Cancerología, Bogotá, D.C, Colombia; 4 Head and Neck Surgery Unit, Instituto Nacional de Cancerología, Bogotá, D. C, Colombia; 5 Faculty of Medicine, Universidad Cooperativa de Colombia, Villavicencio, Colombia

**Keywords:** *BRCA1*, extended phenotype, HBOC syndrome, homologous recombination deficiency, salivary gland tumor

## Abstract

**Background:**

Pathogenic *BRCA1* variants are established in hereditary breast and ovarian cancer (HBOC) and associated with pancreatic, prostate, and gastric cancers. Salivary gland tumors (SGTs) have been reported in *BRCA1/2* carriers and suggested as part of an extended HBOC phenotype based on epidemiological associations. However, functional evidence is lacking, and homologous recombination deficiency (HRD)—the hallmark of BRCA-driven cancers—has not been systematically assessed in *BRCA1*-associated SGTs.

**Case presentation:**

We report a Colombian family segregating the *BRCA1* c.3331_3334delCAAG (p.Gln1111Asnfs*5) founder variant with phenotypic variability across four generations: gastric (31%), breast (37.5%), colorectal (19%), and thyroid cancers (12.5%). The proband, a 61-year-old woman, developed high-grade mucoepidermoid carcinoma of the parotid gland. Germline testing confirmed the familial BRCA1 variant. Tumor profiling revealed the same BRCA1 variant (VAF 56%) plus a pathogenic TP53 mutation (c.730G>T, p.Gly244Cys; VAF 32%), without *BRCA1* loss of heterozygosity. HRD testing using shallow whole genome sequencing showed preserved homologous recombination function (Genomic Instability Score: 0.01, LGA: 11.40, LPC: 0), all below HRD-positive thresholds.

**Conclusion:**

This represents the first SGT in a *BRCA1* carrier evaluated with HRD testing. The absence of HRD argues against BRCA1-driven tumorigenesis despite clear familial segregation. These findings challenge the presumed causal relationship between BRCA1 variants and SGT development. Clinical implications are direct: SGTs in *BRCA1* carriers should not be assumed eligible for PARP inhibitor therapy without HRD confirmation, and enhanced surveillance appears unwarranted. This case underscores that co-occurrence does not establish causation and highlights the critical importance of functional validation before expanding hereditary cancer spectra.

## Introduction

The progressive reduction in sequencing technology costs has facilitated its incorporation into clinical practice, especially for evaluating patients with suspected hereditary cancer predisposition (1). *BRCA1* and *BRCA2* are high-penetrance genes and the main markers for HBOC, with well-established associations with breast, ovarian, pancreatic, prostate, gastric cancers, and melanoma (2–4).

Recently, potential associations between *BRCA1/2* variants and less common neoplasms have emerged. Shen et al. reported SGTs in 0.052% of 5,754 *BRCA1/2* variant carriers (p<0.001) (5), with subsequent case reports (6, 7). These observations align with extended phenotypes proposed in hereditary cancer syndromes (8) and have been discussed alongside biological plausibility based on shared features between salivary and mammary glands (9–11). However, they still lack functional validation through HRD testing.

Germline investigations have largely focused on epidemiological and association-based studies lacking integrated somatic mutational profiles and functional testing. A comprehensive pan-cancer analysis demonstrated that 27% of tumors in patients with high-penetrance pathogenic germline variants neither represented associated cancer types nor exhibited somatic loss of the wild-type allele, suggesting these variants may not contribute to tumorigenesis even in carriers (12).

A critical gap remains: do rare tumors such as SGTs in *BRCA1* carriers exhibit HRD, the functional hallmark of BRCA1-driven tumorigenesis? In BRCA1-associated cancers, biallelic inactivation typically leads to HRD, resulting in distinctive genomic instability patterns characterized by mutational signature 3 (13), including loss of heterozygosity (LOH), telomeric allelic imbalance (TAI), and large-scale transitions (LST) (14, 15). Notably, while signature 3 is characteristic of BRCA-deficient tumors, head and neck carcinomas more commonly exhibit signatures 1B, 2, 4, and 7 (13). Thus, the presence of a germline variant alone is insufficient to establish oncogenic causation—functional evidence of HRD is necessary to implicate BRCA1 in SGT tumorigenesis.

Without functional confirmation, it is not possible to determine whether SGTs in *BRCA1* carriers represent true BRCA-driven malignancies or incidental findings. Here, we present the comprehensive clinical and molecular characterization of a high-grade mucoepidermoid carcinoma of the parotid gland in a *BRCA1* c.3331_3334delCAAG (p.Gln1111Asnfs*5) carrier. By integrating germline, somatic, and HRD analyses, we aimed to assess the potential role of *BRCA1* in salivary gland tumorigenesis and to refine the boundaries of the extended HBOC phenotype.

## Case presentation

### Clinical presentation and histopathology

**Figure 1:**
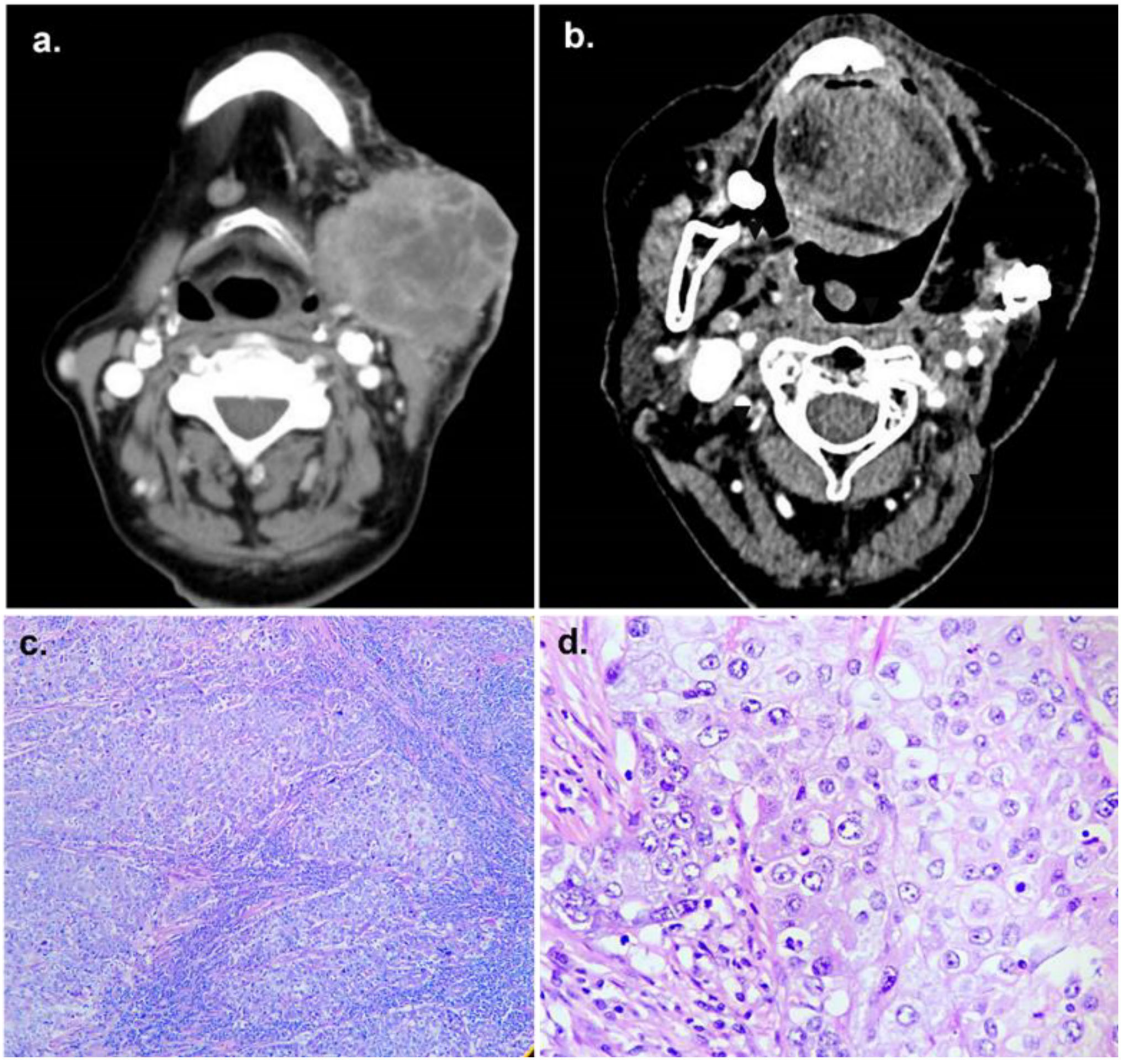
Computerized tomography of paranasal sinuses and H&E histopathology images. **(a)** Mass in the left submandibular region, measuring 61 x 48 mm, with necrosis and skin involvement, causing cortical disruption of the mandibular angle. **(b)** Post-surgical resection of the left mandibular ramus with reconstruction and partial resection of the hypopharyngeal, submandibular, and left parotid regions, showing no lymph node relapse. **(c)** At 4x magnification, H&E stain reveals a malignant epithelial tumor with eosinophilic cytoplasm, pleomorphic nuclei, nests, and dense lymphocytic stroma. **(d)** At 40x magnification, H&E stain shows cells with defined membranes, vesicular chromatin, and visible nucleoli.

The proband, a 61-year-old woman, was referred to the Head and Neck Surgery Unit at the National Institute of Cancerology, Colombia, for evaluation of a progressively enlarging left parotid mass associated with otalgia, limited mouth opening, and weight loss over five months. Paranasal sinus computed tomography revealed a lesion confined to the left parotid gland, extending to adjacent subcutaneous and submandibular tissues, with suspicious ipsilateral lymphadenopathy and no bony infiltration (**Figure 1A**).

Histopathological examination showed a high-grade carcinoma with squamous differentiation (**Figures 1C, D**). Immunohistochemistry was positive for cytokeratin (CK) AE1/AE3, epithelial membrane antigen (EMA), CK5/6, P63, and CK7, with focal CEA expression; CK20 and androgen receptors were negative. The Ki-67 proliferation index was 80%. These findings supported the diagnosis of high-grade mucoepidermoid carcinoma of the parotid gland.

**Figure 2:**
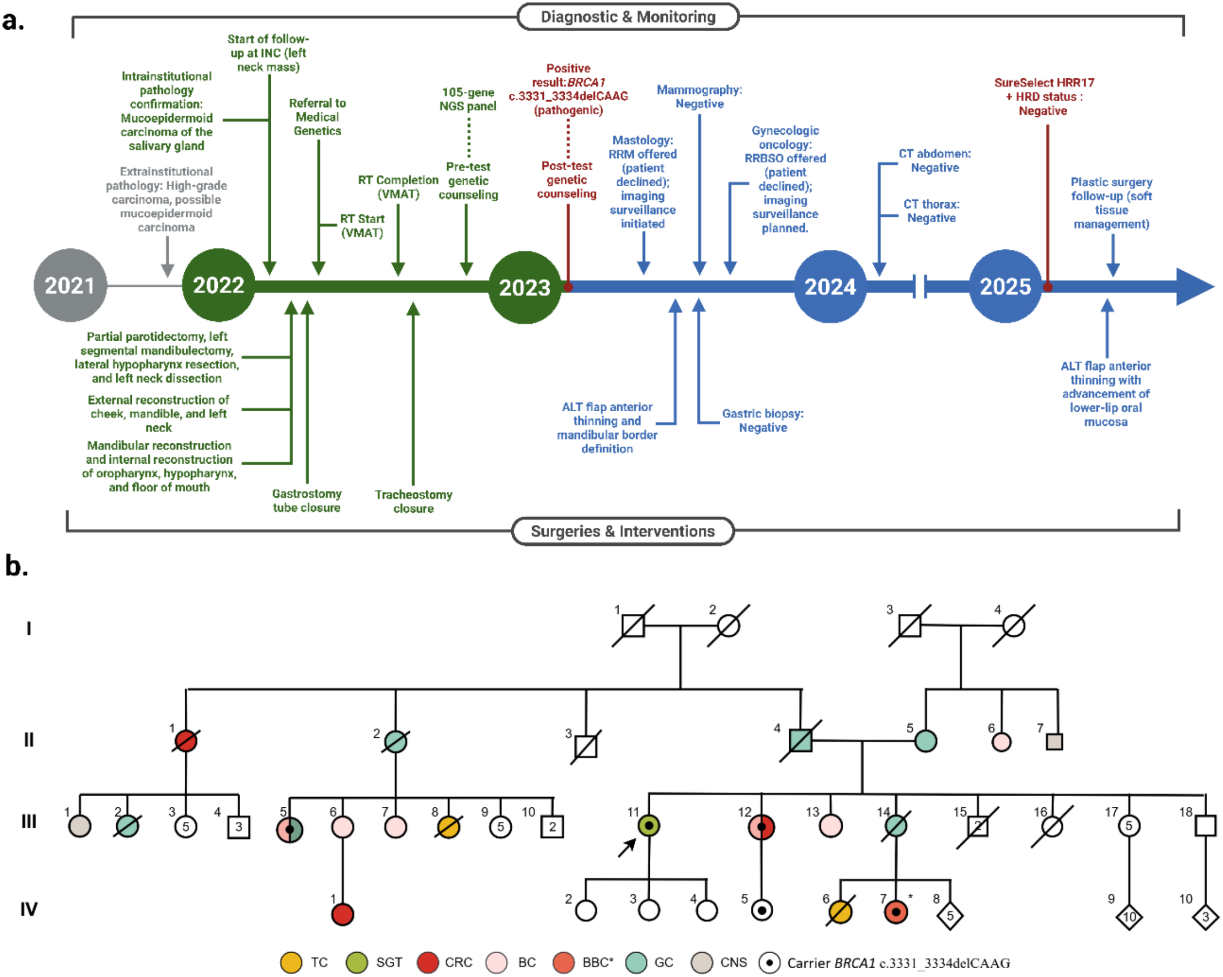
Clinical timeline and family pedigree. **(a)** Timeline of Diagnostic & Monitoring and Surgical & Interventional events for the index case. Gray indicates extrainstitutional clinical procedures; Green, intrainstitutional clinical procedures and oncology follow-up; Blue, surveillance and clinical management after BRCA1 identification; Red, molecular and genetic testing. **(b)** Pedigree showing the oncological history of a Colombian family carrying the familial variant *BRCA1* c.3331_3334delCAAG (p.Gln1111Asnfs*5). The index case is indicated with an arrow. BC, Breast Cancer; BBC*, Bilateral Breast Cancer; CNS, Cancer Not Specified; CRC, Colorectal Cancer; GC, Gastric Cancer; SGT, Salivary Gland Tumor; TC, Thyroid Cancer; ALT flap, Anterolateral Thigh Flap; CT, Computed Tomography; HRD, Homologous Recombination Deficiency; INC, Instituto Nacional de Cancerología (National Institute of Cancerology); NGS, Next-Generation Sequencing; RT, Radiotherapy; RRM, Risk-Reducing Mastectomy; RRBSO, Risk-Reducing Bilateral Salpingo-Oophorectomy; VMAT, Volumetric Modulated Arc Therapy.

The patient underwent partial parotidectomy, segmental mandibulectomy, lateral hypopharyngectomy, left-sided neck dissection, and tracheostomy, followed by fibula flap reconstruction (**Figure 1B**). No perioperative complications were reported. The postoperative evolution, including multidisciplinary follow-up, imaging assessments, and reconstructive evaluations, is summarized in **Figure 2A**. Adjuvant chemotherapy or radiotherapy were not administered; current follow-up shows no evidence of recurrence or new suspicious lesions.

### Family history and germline findings

The family, originating from Huila in southwestern Colombia, demonstrated striking cancer aggregation across four generations (**Figure 2B**, **Table 1**). Among 16 at-risk relatives, malignancies included gastric (5/16, 31%), breast (6/16, 37.5%), colorectal (3/16, 19%), and thyroid cancers (2/16, 12.5%), with two members developing metachronous tumors.

**Table 1:** Clinical and molecular characterization of cancer cases in a Colombian family carrying the *BRCA1* c.3331_3334delCAAG founder variant.

ID	Type cancer	Age to onset	*BRCA1* variant	Relation to proband
Proband	SCG	62	+	–
III12	Breast	52	+	1st degree (sister)
	Colorectal	66	+	
III13	Breast	65	N	1st degree (sister)
III14	Gastric	82	N	1st degree (sister)
II4	Gastric	72	N	1st degree (father)
IV	Thyroid	51	N	2d degree
IV7	Breast (Bilateral)	57	N	2d degree
II1	Colorectal	60-70*	N	3rd degree
II2	Gastric	50-60*	N	3rd degree
III1	Cancer non-specified	>50*	N	4th degree
III2	Gastric	40	N	4th degree
III5	Gastric	48	+	4th degree
	Breast	50	+	
III6	Breast	>50*	N	4th degree
III7	Breast	>50*	N	4th degree
III8	Thyroid	70	N	4th degree
IV1	Colorectal	37	N	5th degree

(+) Confirmed BRCA1 c.3331_3334delCAAG variant carrier by germline molecular testing; (N) Not tested or confirmed non-carrier; (*) Approximate age at diagnosis based on family report. SGC, salivary gland carcinoma; Degree of relation refers to genetic relationship to the proband (1st degree: parents/siblings; 2nd degree: grandparents/aunts/uncles; 3rd-5th degree: cousins of varying distance).

The *BRCA1* variant was first identified in a paternal cousin (III-5) diagnosed with gastric cancer at 48 years and unilateral breast cancer at 50 years, who underwent the MyRisk^®^ Hereditary Cancer Test (Myriad Genetics), revealing a heterozygous pathogenic *BRCA1* frameshift variant c.3331_3334delCAAG; p.Gln1111Asnfs*5. The same variant was later confirmed in the proband’s sister (III-12), affected by unilateral breast cancer at 52 years and colorectal cancer at 66 years.

The proband underwent multigene panel testing (TruSight Cancer – Illumina, 105 genes), which confirmed the same *BRCA1* pathogenic variant, establishing the diagnosis of HBOC syndrome. Cascade testing identified two additional carriers (IV-5 and IV-7), one of whom developed bilateral breast cancer during follow-up (**Table 1**). The presence of the variant in multiple affected relatives demonstrates clear familial segregation consistent with HBOC. After two years of surveillance, the proband has not developed other HBOC-related tumors, Risk-reducing options, including prophylactic mastectomy and bilateral risk-reducing salpingo-oophorectomy, were thoroughly discussed with the patient. However, she ultimately declined to pursue these surgical procedures. Surveillance is currently maintained through close clinical and imaging follow-up, strictly adhering to the established NCCN guidelines.

### Somatic molecular and HRD analysis

To explore the possible contribution of the *BRCA1* variant to salivary gland tumorigenesis, tumor tissue underwent analysis with the SureSelect HRR17 Custom Design panel (Agilent Technologies), which sequences 17 homologous recombination repair (HRR) genes (*BRCA1, BRCA2, ARID1A, ATM, BRAF, BRIP1, CDK12, CHEK1, CHEK2, FANCA, FANCL, NBN, PALB2, PIK3CA, RAD51C, RAD51D, TP53*, and *ZNF276*) and evaluates HRD status using shallow whole-genome sequencing (sWGS).

Genomic instability was quantified using the Large Genomic Alteration (LGA) score and the Loss of Parental Copy (LPC) score. LGA represents the number of breakpoint copies, where a breakpoint corresponds to a change in copy number between two genomic segments of at least 10 Mb in length and separated by no more than 3 Mb. LPC is defined as the number of haploid segments of at least 10 Mb in length, indicating LOH (14). This assay has demonstrated high concordance with the Myriad myChoice™ HRD test (16). Sequencing was performed on an Illumina NextSeq500/550 platform with bioinformatic analysis by the SeqOne^®^ platform.

Tumor analysis for HRR genes confirmed the same germline *BRCA1* pathogenic variant (variant allele frequency [VAF] 56%) and revealed an additional pathogenic somatic *TP53* likely pathogenic variant (c.730G>T, p.Gly244Cys; VAF 32%), absent in the germline. HRD status analysis showed a Genomic Instability Score (GIS) of 0.01, LGA score of 11.40, and LPC score of 0.

The results obtained fall below the established positivity thresholds for sGWG-based HRD technology, according to the criteria defined in the Phase III PAOLA-1/ENGOT-ov25 clinical trial, which serves as the gold standard for validating the clinical efficacy of sGWG-based HRD determination in ovarian cancer (16). The trial categorizes results as (i) definitively negative (non-HRD) for an LGA-score below 18, (ii) borderline for scores between 18 and 22, and (iii) definitively positive (HRD) for an LGA-score exceeding 22, with a central validated cutoff point of 20 for positivity. Furthermore, both the GIS and LPC parameter exhibit clearly negative values, thereby reinforcing the global interpretation of an absence of significant genomic instability.

Given the hypothesis of potential reversion events in *BRCA1/2* tumors lacking HRD, we conducted a bioinformatic analysis using a methodology similar to Murciano-Goroff et al., 2022 (17). BAM and VCF files from the sWGS-based HRD assay were aligned to the hg19 reference genome. Allele-specific copy number states and CNVs were inferred with FACETS v0.6.0 and facets-suite v2.0.6, and mutational signatures were evaluated with the mutation-signatures R package. No evidence of reversion variants, secondary biallelic inactivation, or pathogenic CNVs was identified.

### Literature review

A comprehensive literature review was conducted through March 2025 using MEDLINE/PubMed, EMBASE, and SCOPUS databases. The search strategy combined terms for salivary gland tumors (“mucoepidermoid carcinoma”, “salivary gland carcinoma”) with “*BRCA1*”, “*BRCA2*”, and “homologous recombination deficiency” in both germline and somatic contexts. Eligible articles included cases, series, cohort studies, or functional analyses linking *BRCA1/2* germline variants with salivary gland neoplasms or evaluating HRD status in these tumors.

## Discussion

This case illustrates the phenotypic variability of the *BRCA1* c.3331_3334delCAAG (p.Gln1111Asnfs*5) founder variant, a recurrent allele in Andean populations (7, 18–20). In this Colombian family, affected members developed gastric (31%), breast (37.5%), colorectal (19%), and thyroid cancers (12.5%), demonstrating an atypical HBOC distribution (**Table 1**). Among the four confirmed carriers, the diversity was striking: gastric and breast cancer (III-5), breast and colorectal cancer (III-12), bilateral breast cancer (IV-7), and, uniquely, a high-grade mucoepidermoid carcinoma of the parotid gland in the proband. This spectrum aligns with prior reports suggesting that Latin American *BRCA1* variants may confer distinct risks, particularly for gastric cancer (4, 21–26).

Salivary gland tumors are rare, accounting for only 3% to 10% of all head and neck tumors (27). They are predominantly sporadic and are generally not associated with hereditary cancer syndromes (28). Within this context, the occurrence of an SGT in a *BRCA1* carrier is particularly uncommon and raises the possibility of an expanded phenotype. Supporting this hypothesis, salivary and mammary glands share embryological origin, tubuloacinar exocrine architecture, and overlapping morphological and immunohistochemical features (9). Both tissues express markers such as CK5/6, P63, and EMA—all present in our patient’s tumor—and display pathological similarities, including sclerosing polycystic adenosis as a counterpart of fibrocystic breast changes (10). Furthermore, salivary duct carcinomas with *ERBB2* amplification have demonstrated clinical responses to trastuzumab, mirroring HER2-positive breast cancer (29, 30). These parallels have supported the rationale proposed by Shen et al. (5), De Barros et al. (6), and Ripamonti et al. (11), who suggested *BRCA1/2* variants might contribute to SGT pathogenesis.

However, biological plausibility and epidemiological associations cannot establish causality. Our case contributes evidence by integrating HRD testing, a functional biomarker of BRCA-related oncogenesis. Despite the presence of the familial BRCA1 loss-of-function variant, the tumor retained homologous recombination proficiency. No *BRCA1* LOH was detected, and instead, a somatic *TP53* variant (p.Gly244Cys) was identified as a more likely oncogenic driver. Variants in TP53 are observed in various sporadic cancers including a large proportion of head and neck cancers (31), and *TP53*—along with *CDKN2A*—represents one of the most common somatic alterations in high-grade mucoepidermoid carcinomas (9, 31). These findings argue strongly against BRCA1-driven tumorigenesis in this SGT.

Our findings contrast with previous reports suggesting *BRCA1*–SGT associations based on epidemiological signals or partial molecular data. The literature review identified one epidemiological study (5), two case reports describing three patients (6, 11), and a pan-cancer cohort study reporting two additional cases with *BRCA1* germline variants (12). Shen et al.’s analysis (5) provided the first statistical signal, while Ripamonti et al. (11) documented LOH in one *BRCA1* carrier; however, none of these studies included HRD testing—the definitive marker of BRCA-driven tumorigenesis. De Barros et al. (6) reported a single case with only germline testing, without somatic or functional characterization. This reliance on co-occurrence or LOH alone illustrates a methodological gap: LOH may represent one mechanism of biallelic inactivation, but, as our case shows, its presence (or absence) does not determine HRD status. Without functional validation, causal inference remains speculative.

Broader observational data reinforces this interpretation. In a pan-cancer cohort of 149 salivary gland carcinomas, two tumors carried a *BRCA1* frameshift variant (p.Glu23Valfs*17), yet independent HRD testing demonstrated homologous recombination proficiency (median LST ≈ 10; median AI ≈ 5; median LOH% ≈ 0.5) (12). These findings, together with the current case report, reinforce the concept that SGTs occurring in individuals with *BRCA1* variants are not necessarily indicative of BRCA1-induced malignancy. While these cases demonstrate SGTs with *BRCA1* variants and negative HRD status, the available clinical information is insufficient. Specifically, it is unclear whether the donors had a diagnosis of HBOC syndrome or whether the VAF suggested the presence of these variants in germline.

In this context, the present case stands out as the first to establish an association between clinical diagnosis, family history, somatic molecular profiling, and HRD testing, providing a more comprehensive understanding of SGTs in *BRCA1* carriers (see **Table 2**).

**Table 2:** Summary of published studies reporting salivary gland tumors in BRCA1/2 variant carriers, highlighting the evolution from epidemiological associations to functional validation.

Year	Gene	Study type	Cases/Population	Statistical significance	Clinical and molecular characteristics	HRD/Genomic assessment	Ref
2014	*BRCA1/2*	Population-based	3/5,754 carriers (0.052%) vs 0.003% general population	p<0.001	No clinical or molecular characterization	Not performed	Shen et al. (5)
2020	*BRCA1*	Case report	1 patient	N/A	Female, 55 years; MEC; *BRCA1* p.Ser157fs*1	Not performed	De Barros et al. (6)
2021	*BRCA1/2*	Case series	5 families (2 with molecular testing)	N/A	Two cases (55 and 65 years); *BRCA1* p.Ser267Lysfs19; *BRCA2* p.Lys3161	LOH detected; HRD not tested	Ripamonti et al. (11)
2021	*BRCA1*	Pan-cancer observational cohort	2/149 SGC with *BRCA1* variants	N/A	*BRCA1* p.Glu23Valfs*17; Median age: 59 years	HRD negative (Median LST = 10, AI = 5, LOH%=0.5)	Srinivasan et al. (12)
2025	*BRCA1*	Case report	1 patient	N/A	Female, 61 years; High-grade MEC; *BRCA1* p.Gln1111Asnfs*5	HRD negative (GIS: 0.01, LGA: 11.40, LPC: 0)	Current study

EC, mucoepidermoid carcinoma; SGC, salivary gland carcinoma; LOH, loss of heterozygosity; HRD, homologous recombination deficiency; GIS, Genomic Instability Score; LGA, Large Genomic Alteration; LPC, Loss of Parental Copy; LST, Large-scale State Transitions; AI, Allelic Imbalance; N/A, not applicable.

Alternative mechanisms have been proposed for BRCA-related oncogenesis, including dominant-negative effects of truncated proteins (32) or metabolic interference with *BRCA1/2* function (33). Yet, all ultimately converge on HRD. Moreover, based on the reversion hypothesis (17), a dedicated bioinformatic analysis was conducted for this purpose, but no reversion variants, additional biallelic inactivation, or pathogenic CNVs were detected. The absence of HRD in our patient therefore provides strong evidence that the tumor arose independently of BRCA1 dysfunction.

Clinically, the HRD-negative status has direct implications. HRD-positive tumors show superior responses to PARP inhibitors across multiple malignancies, while HRD-negative tumors derive minimal benefit (34–37). In our case, the HRD-negative status cautions against using PARP inhibitors in this setting and underscores the importance of functional validation before extrapolating targeted strategies beyond tumor types with established BRCA dependence. While the statistical signal reported by Shen et al. suggests that SGTs can occur in BRCA families (5), the absolute risk remains very low, and our data do not support enhanced surveillance for salivary glands in *BRCA1* carriers at this time.

This case illustrates a broader challenge in contemporary oncology genetics: as multigene testing expands, clinicians will encounter pathogenic germline variants in tumors outside classical spectra. In such contexts, integrating germline and somatic profiles with functional biomarkers is essential to discriminate coincidental co-occurrence from causal associations—particularly relevant in Latin America, where founder effects and population structure may shape distinct phenotypic patterns (18–25). Future studies in diverse cohorts, combining HRD assays with detailed clinicopathologic annotation, will be key to refining the true boundaries of the BRCA1-associated phenotype and to aligning surveillance and therapy with underlying tumor biology.

## Conclusion

We present the first salivary gland tumor in a *BRCA1* c.3331_3334delCAAG carrier evaluated with functional HRD testing. Despite clear familial segregation of this pathogenic variant in a Colombian family with diverse cancers, the proband’s high-grade mucoepidermoid carcinoma showed preserved homologous recombination repair, indicating that BRCA1 inactivation was not the primary oncogenic driver.

These findings, combined with recent evidence of absent HRD in other *BRCA1*-associated SGTs (12), argue against including SGTs within the HBOC spectrum without molecular validation. Our results have direct clinical implications: SGTs diagnosed in individuals carrying a *BRCA1* pathogenic variant should not be automatically presumed eligible for PARP inhibitor therapy or other treatments that exploit HRR weakness for their cytotoxic effect. Furthermore, in such cases, enhanced surveillance appears unwarranted based on HRD status alone. This clinical observation underscores that the co-occurrence of a hereditary mutation and a tumor type does not establish a causal relationship. It crucially highlights the importance of performing functional validation before expanding hereditary cancer spectra or selecting targeted therapies.

## Limitations

This manuscript constitutes a single case report. While this specific finding demonstrates unequivocal HRD negativity in a SGT and provides powerful evidence to question the necessity of routinely including SGTs within the established HBOC syndrome spectrum for HRD-driven therapy, it alone cannot definitively refute the potential association. We therefore strongly encourage future studies to perform HRD testing on a larger cohort of patients with SGT. Furthermore, it is important to note that the HRD analysis in this case was performed using sWGS, a technique which is not yet widely adopted as an official companion diagnostic and utilizes different scoring cutoffs compared to other established approaches.

## Data Availability

The original contributions presented in the study are included in the article. Further inquiries can be directed to the corresponding author.
